# Local Knowledge and Human–Wildlife Conflict in the Conservation of the Harpy Eagle ( *Harpia harpyja* ) in Its Main Refuge in the Atlantic Forest

**DOI:** 10.1002/ece3.73499

**Published:** 2026-05-12

**Authors:** Brener Fabres, Tânia Margarete Sanaiotti, Gustavo Magnago, Gabriel Scaldaferro Bonfa, Henrique Mariano, Paulo Quadros de Menezes, José Alves da Costa Filho, Mylena Kaizer, Carlos Hartur Ribeiro Nóia, Frederico Pereira de Castro Andrade, Francisca Helena Aguiar‐Silva, Olivier Jaudoin, Ana Carolina Srbek‐Araujo, Geovane Souza Siqueira, Aureo Banhos

**Affiliations:** ^1^ Programa de Pós‐Graduação em Ciências Biológicas‐Biologia Animal – PPGBAN, Universidade Federal do Espírito Santo ‐ UFES Vitória Espírito Santo Brazil; ^2^ Projeto Harpia Mata Atlântica (Atlantic Forest Harpy Eagle Project), Universidade Federal do Espírito Santo ‐ UFES Vitória Espírito Santo Brazil; ^3^ Projeto Harpia (Harpy Eagle Project) Instituto Nacional de Pesquisas da Amazônia – INPA Manaus Amazonas Brazil; ^4^ Coordenação de Biodiversidade, Instituto Nacional de Pesquisas da Amazônia – INPA Manaus Amazonas Brazil; ^5^ Laboratório de Ecologia e Conservação de Biodiversidade, Programa de Pós‐Graduação em Ciência Animal Universidade Vila Velha Vila Velha Espírito Santo Brazil; ^6^ Reserva Natural Vale Linhares Espírito Santo Brazil; ^7^ Departamento de Biologia, Centro de Ciências Exatas, Naturais e da Saúde Universidade Federal do Espírito Santo ‐ UFES Alegre Espírito Santo Brazil

**Keywords:** birdwatching, citizen science, coexistence, protected areas, threatened species

## Abstract

The Harpy Eagle ( *Harpia harpyja* ), one of the largest and most powerful raptors in the world, inhabits tropical forests of the Americas and is currently threatened with extinction throughout its range. In this study, we analyzed occurrence records of the species over a 50‐year period in the largest remaining portion of Tabuleiro Forest within the Atlantic Forest biome, in southeastern Brazil, one of the most threatened biodiversity hotspots in the world. We compiled 88 records, including 44 adults, eight pairs, eight juveniles, 19 individuals of undetermined age class, and nine nests. Additionally, we documented at least 73 historical and recent records from surrounding areas. The records were contributed by researchers, reserve staff, birdwatchers, and local residents, underscoring the role of citizen science in monitoring. Among the recorded individuals, one adult and one juvenile were shot, two juveniles died from electrocution, and one adult was roadkilled, revealing persistent human–wildlife conflict. This study highlights the contributions of local communities to species monitoring and the urgent need to mitigate anthropogenic threats. Promoting coexistence through public engagement and conservation strategies is essential to ensure the long‐term survival of the Harpy Eagle in its last reproductive refuges within the Atlantic Forest.

## Graphical abstract

The Harpy Eagle ( *Harpia harpyja* ), a top predator of Neotropical forests, faces severe threats across its range, including in the Atlantic Forest. Using 50 years of occurrence records, including nests and multiple age classes, we document the species' persistence in one of its last reproductive refuges while revealing ongoing human–wildlife conflicts such as persecution, electrocution, and roadkill. Our findings underscore the vital role of local knowledge and citizen science in monitoring and highlight the urgent need for conservation strategies to promote coexistence.

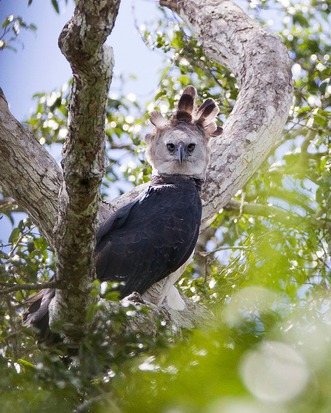

## 1 Introduction

**FIGURE 1:**
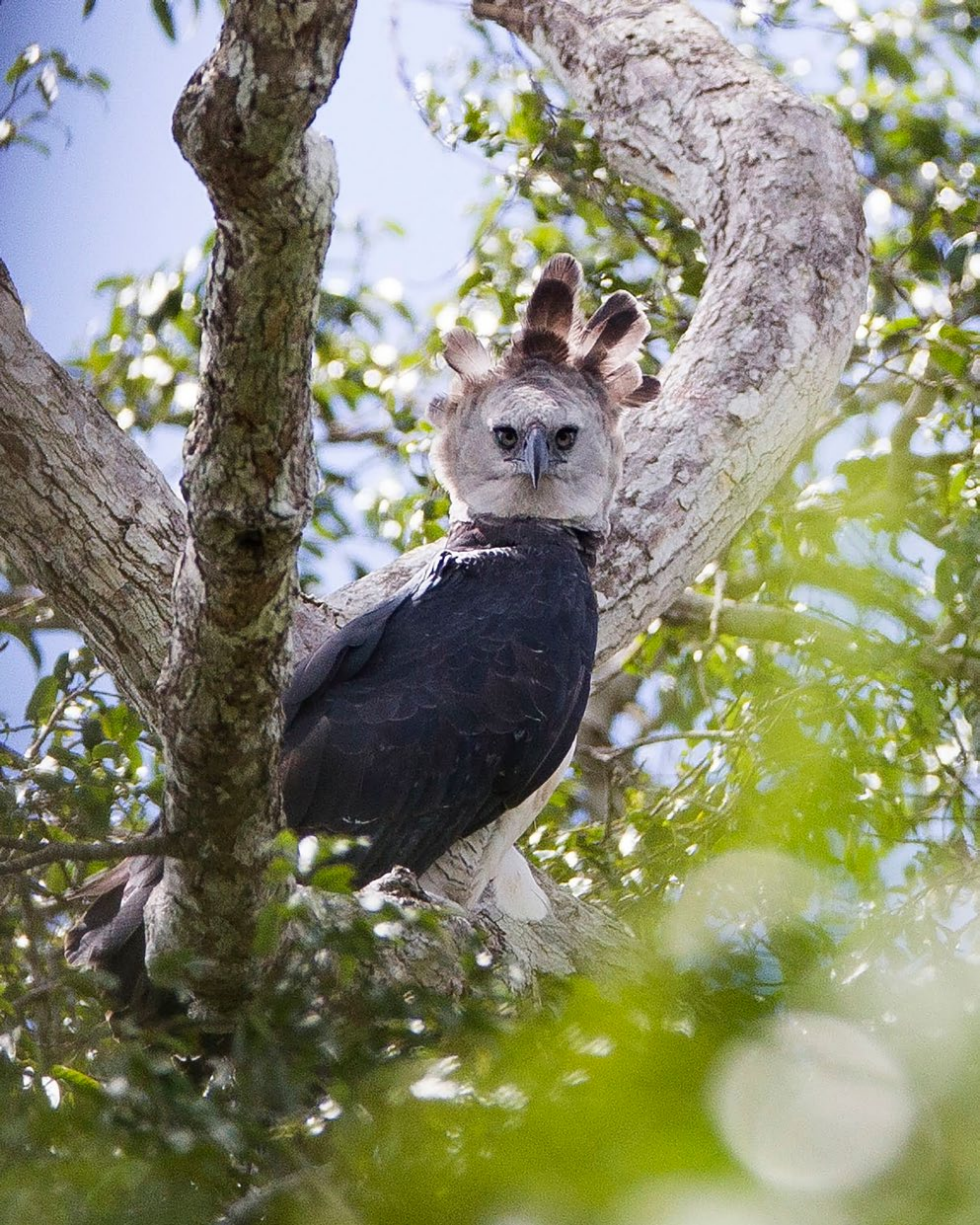
*Harpia harpyja* in the Vale Natural Reserve. Photo by João Marcos Rosa, 15 March 2017.

The Harpy Eagle ( *Harpia harpyja* ) (Figure 1) is one of the largest and most powerful eagles in the world, with wingspans exceeding 2 meters (Conway 1962; Fowler and Cope 1964; Aguiar‐Silva et al. 2023). Its range spans Neotropical forests from southern Mexico to northeastern Argentina, with broad distribution in Brazilian forests, particularly the Amazon and the Atlantic Forest (Banhos et al. 2018; Sutton et al. 2021; BirdLife International 2022). The species depends on large forest remnants for its survival (Sutton et al. 2021; Vargas et al. 2006), feeding primarily on arboreal mammals such as sloths and monkeys (Piana 2007; Aguiar‐Silva et al. 2014; Kaizer et al. 2023), and nesting in emergent trees in the forest canopy (Rettig 1978; Luz 2005; Giudice et al. 2007; Miranda et al. 2020).

The Harpy Eagle is classified as Vulnerable to extinction throughout its geographic range (BirdLife International 2022), including in Brazil (Banhos et al. 2018; Ministério do Meio Ambiente 2022). Given its large home range requirements in forested landscapes (Vargas et al. 2006), habitat loss is one of the main threats to its conservation (BirdLife International 2022; Miranda, Peres, Olmos, et al. 2021). In addition, the species faces pressures from poaching for consumption and arbitrary persecution often driven by human curiosity (de Freitas et al. 2014; Trinca et al. 2008; Giraldo‐Amaya et al. 2021; Miranda, Peres, and Downs 2021). Individuals are also removed from the wild due to socio‐environmental conflicts, some are killed by vehicle collisions (Banhos et al. 2015) or electrocution on power lines (Gusmão et al. 2020), while others are captured alive, either rescued or confiscated by environmental authorities, and transferred to captive breeding facilities and zoos (de Oliveira et al. 2022).

Despite these threats, the Harpy Eagle is also regarded as a charismatic and flagship species for biodiversity conservation. Its image has been strategically used in educational programs aimed at ecosystem and territory preservation (Curti and Valdez 2009). Moreover, its nesting sites offer strong potential for promoting local ecotourism (Miranda et al. 2022), contributing to regional sustainable development.

Among the biomes where the Harpy Eagle occurs, the Atlantic Forest hosts the largest human population (Fundação SOS Mata Atlântica and Instituto Nacional de Pesquisas Espaciais 2023). This biome is one of the most threatened biodiversity hotspots in the world (Mittermeier et al. 2004), is currently highly fragmented, with only 11.4%–16% of its original vegetation cover (Ribeiro et al. 2009). In this context, the Harpy Eagle is considered Critically Endangered within the biome (Bergallo et al. 2000; Bressan et al. 2009; Chaves et al. 2019; Machado et al. 2005; Bencke et al. 2003; Mikich and Bérnils 2004; Secretaria do Meio Ambiente – SEMA 2017). Numerous records of the species in the wild have been documented throughout the Atlantic Forest (Kaizer et al. 2023; Banhos et al. 2015; Ruschi 1954; Ruschi 1965; Ruschi 1969; Ruschi 1977; Ruschi 1979; Instituto Brasileiro de Desenvolvimento Florestal ‐ IBDF 1981; Peixoto and Peixoto 1986; Chebez et al. 1989; Albuquerque 1995; de Lucca 1996; Galetti et al. 1997; Sick 1997; Pacheco et al. 2003; Srbek‐Araujo and Chiarello 2006; Luz et al. 2005; Anfuso et al. 2008; Novaes et al. 2010; Sánchez‐Lalinde et al. 2019; Aguiar‐Silva et al. 2012; Raton 2012; Meller and Guadagnin 2016; Magnago 2018; Ramaldes 2026; de Menezes et al. 2024). However, conflicts between humans and Harpy Eagles have also been reported (Banhos et al. 2015; Ruschi 1979; Meller and Guadagnin 2016), and some specimens housed in museum collections (Ruschi 1979; Banhos et al. 2016; Banhos et al. 2023), zoos and captive breeding centers (de Oliveira et al. 2022) are victims of such conflicts within the biome.

One of the regions with the highest concentration of Harpy Eagle records is a forest remnant in the northern part of the Brazilian state of Espírito Santo (Kaizer et al. 2023; Banhos et al. 2015; Ruschi 1954; Ruschi 1965; Ruschi 1969; Ruschi 1977; Ruschi 1979; Instituto Brasileiro de Desenvolvimento Florestal ‐ IBDF 1981; Peixoto and Peixoto 1986; Galetti et al. 1997; Sick 1997; Pacheco et al. 2003; Srbek‐Araujo and Chiarello 2006; Novaes et al. 2010; Aguiar‐Silva et al. 2012; Raton 2012; Magnago 2018; Ramaldes 2026), surrounded by a densely populated rural community (Instituto Chico Mendes de Conservação da Biodiversidade – ICMBio 2014; Rolim et al. 2016). The Tabuleiro Forest of northern Espírito Santo, together with those in southern Bahia, is part of the Hileia Baiana (Faria et al. 2021), is one of the most biodiverse areas of the Atlantic Forest biome, with ecological characteristics like those of the Amazon Rainforest (Buso Junior et al. 2013), and currently harbors the only known active nests of the species throughout the biome (Kaizer et al. 2023; Luz et al. 2005; Aguiar‐Silva et al. 2012). These findings have resulted from collaborations among researchers, reserve staff, birdwatchers, and local residents (Aguiar‐Silva et al. 2012).

In this study, we compiled Harpy Eagle records collected by the local community over a 50‐year period and demonstrate how these data have contributed to understanding the population of the species in one of its last known reproductive strongholds in the Brazilian Atlantic Forest, despite ongoing socio‐environmental conflicts. We analyzed patterns in these records and highlighted the valuable insights they provide into the species and its local conservation status.

## 2 Materials and Methods

### 2.1 Study Area

**FIGURE 2:**
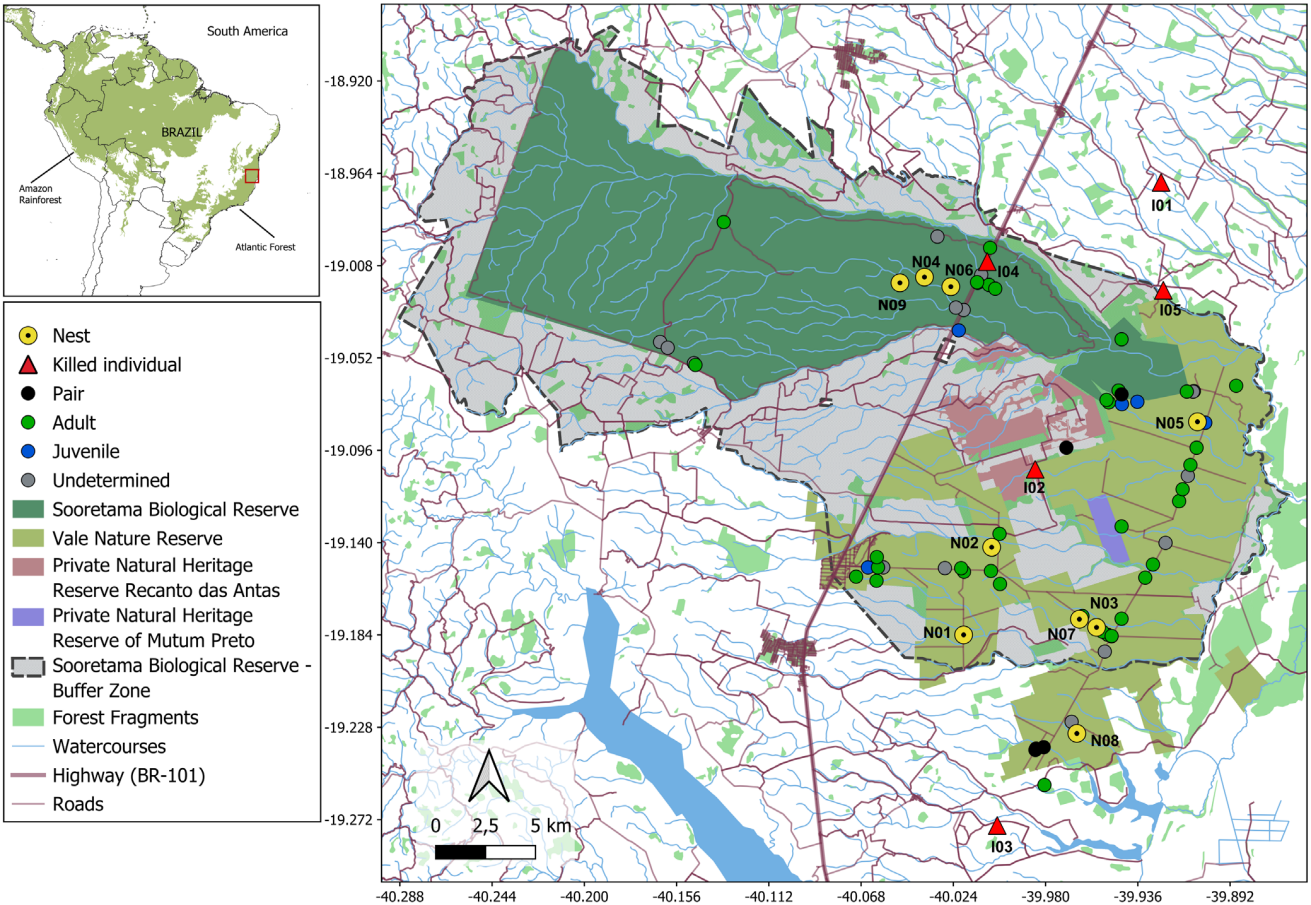
Study area showing the Sooretama Biological Reserve and the Vale Natural Reserve, along with the locations of Harpy Eagle records between the early 1970s and 2026. Nest sites are represented by large yellow circles, labeled with their identification numbers; locations where individuals were found dead are shown as red triangles, also labeled with their identification numbers. The map was created using licensed QGIS Desktop software, version 3.44.3, an open‐source platform for geospatial analysis.

This study was conducted in two contiguous Atlantic Forest fragments, the Vale Natural Reserve (VNR) and the Sooretama Biological Reserve (SBR) (Figure 2), and their surrounding areas, located in the municipalities of Linhares, Sooretama, Jaguaré, and Vila Valério, in the state of Espírito Santo (ES), southeastern Brazil. The SBR is a federally protected area encompassing 27,558 ha (Instituto Chico Mendes de Conservação da Biodiversidade – ICMBio 2014), while VNR is a privately owned reserve covering 22,711 ha (Rolim et al. 2016). The reserves have a long history of conservation, dating back to the 1940s (Instituto Brasileiro de Desenvolvimento Florestal ‐ IBDF 1981; Instituto Chico Mendes de Conservação da Biodiversidade – ICMBio 2014; Rolim et al. 2016). Along with two additional state conservation units, the Mutum Preto and Recanto das Antas Private Natural Heritage Reserves (PNHRs), covering 378 ha and 2201 ha, respectively (Figure 2), these reserves form the largest preserved remnant of the Tabuleiro Atlantic Forest, totaling approximately 53,000 ha (Rolim et al. 2016). Together with six other reserves in southern Bahia (BA), these areas are part of the Discovery Coast Atlantic Forest Reserves, designated as a UNESCO World Heritage Site (United Nations Educational, Scientific and Cultural Organization ‐ UNESCO 1999). This region in ES and BA is within the Hileia Baiana, a subdivision of the Tabuleiro Forest, one of the most threatened phytophysiognomies of Atlantic Forest (Faria et al. 2021).

The reserves are situated within the Doce and Barra Seca river basins. Vegetation types include lowland Tabuleiro forest (with trees reaching over 35 m in height), *muçununga* (white‐sand vegetation), and native grasslands, interspersed with swamps and broad, shallow valleys (Rolim et al. 2016). Annual precipitation ranges from 1300 to 1600 mm, and the climate is classified as tropical with a dry winter (Aw), according to the Köppen system (Alvares et al. 2013). The mean annual temperature is 24.3°C ± 2.1°, with average minimums of 18.7°C ± 0.6° and average maximums of 29.9°C ± 0.9° (Saiter et al. 2017).

### 2.2 Records

We reanalyzed the records compiled by Aguiar‐Silva et al. (2012) up to 2012 for the VNR. Additional records from both VNR and SBR, collected through January 2026, were compiled from reports by reserve staff, local residents, birdwatchers, researchers, and the field teams of the Harpy Eagle Project (until 2016) and the Atlantic Forest Harpy Eagle Project (from 2016 onward). Additional historical and recent records were obtained from municipalities in ES, in the surroundings of the reserves, based on information provided by community members and literature sources, from 1929 to March 2026.

All data were organized in spreadsheets and mapped, with supporting evidence including photographs, direct sightings, vocalizations, shed feathers, and nest records. When possible, each record included information on age class (juvenile or adult), sex (male or female), and potential pair formation, along with the date, geographic coordinates, observer name, occupation and affiliation, and an assigned identification code. The sex of deceased individuals and shed feathers was determined based on the study by Banhos et al. (2023).

These records were also used to guide active nest searches, conducted on foot along forest trails or with the aid of a DJI Mavic 2 Zoom drone. Tree species used for nesting were identified, and their conservation statuses for each species were obtained from the IUCN Red List (International Union for Conservation of Nature – IUCN 2026).

## 3 Results

### 3.1 Records

Between the early 1970s and January 2026, a total of 88 Harpy Eagle records were documented in the forest complex comprising the SBR–VNR and its surrounding areas (Figure 2 and Appendix S1). Of these, 25 had been previously compiled by Aguiar‐Silva et al. (2012): 24 within the VNR and one in the surrounding landscape. Among these records: 19 were obtained from local community members by the original authors (Aguiar‐Silva et al. 2012); one was shared with other authors (Galetti et al. 1997; Pacheco et al. 2003); one was shared between Peixoto and Peixoto (1986) and Pacheco et al. (2003); two were from Galetti et al. (1997); one from Pacheco et al. (2003); and one from Srbek‐Araujo and Chiarello (2006). The remaining 63 records were compiled in the present study: 32 from VNR, 27 from SBR, and four from surrounding areas. Of these, one was reported by IBDF (Instituto Brasileiro de Desenvolvimento Florestal ‐ IBDF 1981), one by Pacheco et al. (2003), one by Banhos et al. (2015), one by Betkowski (2015) and 11 by birdwatchers (Magnago 2015a, 2015b; Barros 2016; Briso 2017; Bonfa 2017; Bonfa 2024a; Bonfa 2024b; Bonfa 2024c; Bonfa 2024d; Bonfa 2025), while the remaining 47, were recorded directly by this study (Appendix S1).

Of the 88 records, 22 were made by VNR staff, 18 by researchers, 13 by the Atlantic Forest Harpy Eagle Project team, 11 by birdwatchers, 11 by SBR staff, seven by local residents near the reserves, three by the Harpy Eagle Project team, two through collaboration between the Harpy Eagle Project and VNR staff, and one through collaboration between VNR staff and researchers (Appendix S1). The records include 30 sightings, 15 photographs, 11 feather collections, 10 vocalization reports, three photographs accompanied by vocal recordings, two sightings with vocal recordings, two fatalities with photographic documentation, two rescue events involving release, death and photographic documentation, one rescue event with release and photographic documentation, one death with photo, one sighting with feather collection, and one occurrence report (Appendix S1). All of these refer to records outside nests. In addition, nine nest discoveries were documented (Appendix S1).

Regarding life stages and social composition of Harpy Eagles in non‐nest records, 44 were adults, eight were adult pairs, eight were juveniles, 19 were individuals of undetermined age, and nine were nests (Figure 2 and Appendix S1). Records were distributed across 11 months of the year, except for October, and were most frequent from March to September (Appendix S1). Individuals were also observed at six of the nine nests upon their discovery: two nests had a single nestling each, and four nests had one adult present. Three nests had no individuals present at the time of discovery.

**FIGURE 3:**
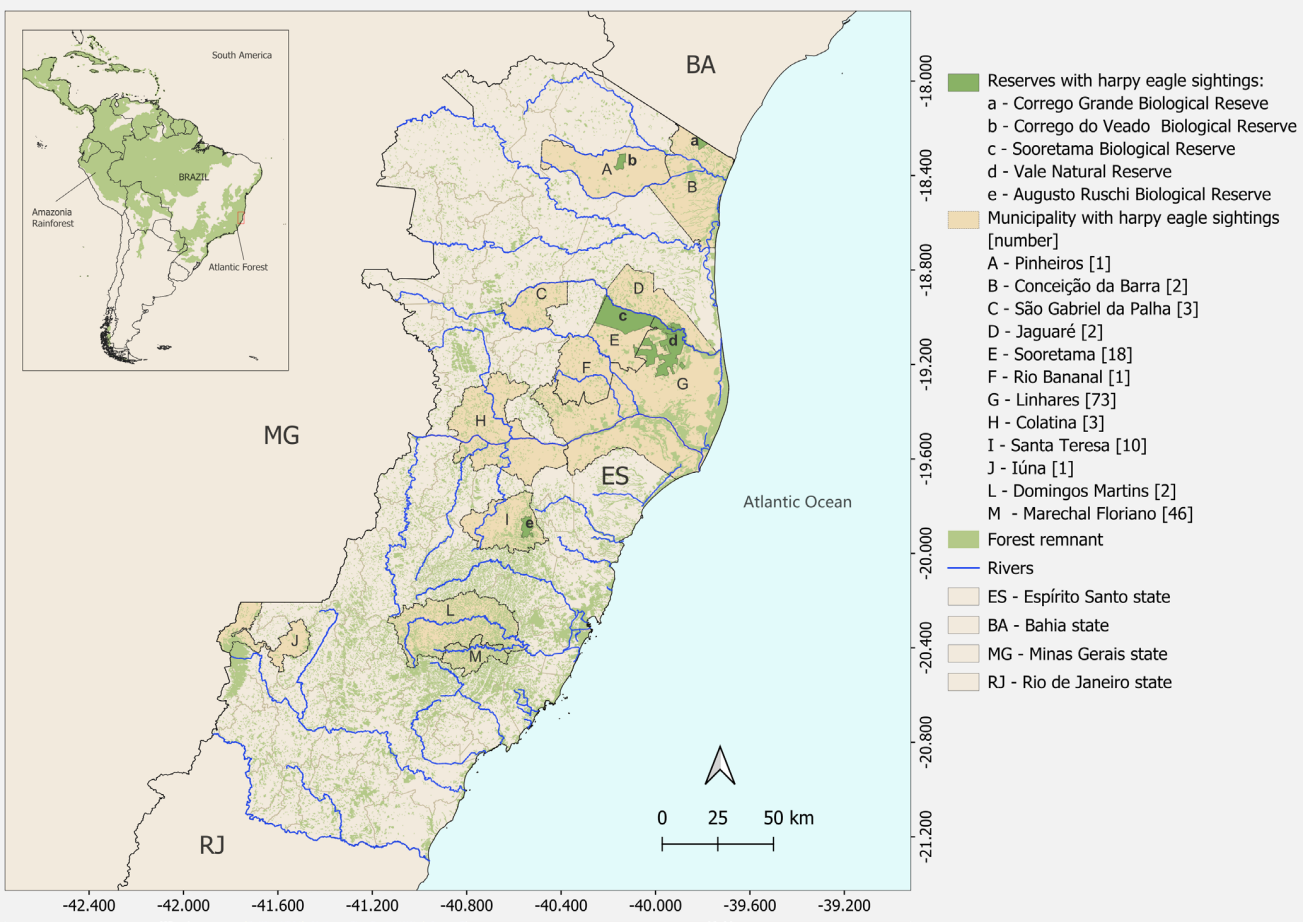
Municipalities and reserves in the state of Espírito Santo, Brazil, with Harpy Eagle records from 1929 to 2026.

Among the municipalities that encompass the SBR–VNR forest complex, 68 records were from Linhares, 18 from Sooretama and two from Jaguaré. We also documented at least 73 additional historical and recent Harpy Eagle occurrence records from other municipalities in ES, dated between 1929 and 2026, many based on information provided by local community members, including four additional historical records of hunting, all located at least 200 km from the SBR–VNR forest complex (Figure 3 and Appendix S2). Of these, five additional historical records were from the municipality of Linhares, located within approximately 50 km of the reserves (Appendix S2). When combined with the records from within the reserves, Linhares accounts for the highest number of Harpy Eagle records in ES, totaling 73 occurrences (Figure 3).

### 3.2 Recorded Nests

Of the nine nests recorded, two had been previously identified (N01 and N02, Figure 2). N01 was located by a researcher in 1992 (Pacheco et al. 2003; Aguiar‐Silva et al. 2012), and an eaglet was present at the time (Bret M. Whitney, personal communication). By 2010, this nest tree had died (Aguiar‐Silva et al. personal communication), and no further breeding activity was recorded at the site. N02 was identified within the VNR in 2010 by Aguiar‐Silva et al. (2012), but only a single egg‐laying was documented in 2019, which was unsuccessful.

In 2016, a new nest was discovered during a birdwatching hike in the RNV (N03, Figure 2). A pair of Harpy Eagles successfully raised an eaglet at this site in 2017, and the nest tree was used until 2021. The nest structure collapsed in 2021 and was not rebuilt by the pair.

In 2017, the Atlantic Forest Harpy Eagle Project team located a nest in the SBR (N04, Figure 2) during a foot survey near a research trail. The nest tree was situated just 250 m from a 2015 sighting of a Harpy Eagle pair by another research team (Betkowski 2015). The nest tree was already dying when the nest was discovered and contained a flightless eaglet that remained in the nest until 2019. Although the tree was dead, the Harpy Eagle pair continued to make reproductive attempts at the site until 2023, without success.

Four additional nests were discovered using drones by the Atlantic Forest Harpy Eagle Project team. In 2019, one was in the VNR (N05, Figure 2) following a 2015 sighting of a juvenile by birdwatchers nearby (Magnago 2015b). The nest was visited by the Harpy Eagle pair until late 2025, all of which were unsuccessful.

In 2021, another nest was located in the SBR (N06, Figure 2) with the aid of a drone, less than 1.430 km from the N04. The Harpy Eagle pair attempted to reproduce at this site until 2022, but without success.

In 2022, a new nest was discovered in the VNR (N07, Figure 2), also using drone assistance, approximately 960 km from the N03. The pair attempted to reproduce at this site through 2024, again unsuccessfully. Individuals maintained the nest in 2025, with visit records until January 2026.

In 2022, another nest was identified in VNR (N08, Figure 2) following a 2019 sighting of a Harpy Eagle pair by Atlantic Forest Harpy Eagle Project researcher, approximately 1 km from the newly discovered nest. The pair attempted to reproduce at this site again in 2024, but once more without success.

In 2026, a new nest was found in the SBR (N09, Figure 2) during a foot survey near a research trail. At the time of discovery, no Harpy Eagle was observed. A pair of Harpy Eagles appeared on camera a few days later carrying branches to refurbish the nest. This nest is located approximately 1.330 km from nest N04 and 2.770 km from nest N06 (Figure 2).

The trees hosting the nine nests were located between 250 m and 5 km from the forest edge adjacent to agricultural areas. Four different tree species were used for nesting: three *Eriotheca macrophylla* (K. Schum.) A. Robyns, 1963 (N03, N06, and N08, Figure 2; 39 m, 40 m, and 38 m tall, respectively), three *Astronium concinnum* Schott ex Spreng., 1827 (N02, N05, and N09, Figure 2; 39 m, 46m, and not measured, respectively), two *Cariniana legalis* (Mart.) Kuntze, 1898 (N01 and N04, Figure 2; both 36 m tall), and one *Manilkara longifolia* (A. DC.) Dubard, 1915 (N07, Figure 2; 40 m tall). Two of these species are globally threatened: *C. legalis* is listed as Endangered and *M. longifolia* as Near Threatened on the IUCN Red List (International Union for Conservation of Nature ‐ IUCN 2026). Notably, both nest trees that died were in *C. legalis* .

### 3.3 Human–Harpy Eagle Conflicts

Poaching (I01 and I05, Figure 2), electrocution (I02 and I03, Figure 2), and roadkill (I04, Figure 2) were the causes of Harpy Eagle deaths documented in this study.

I01 was poached in the late 1970s, likely between 1977 and 1979, in the district of Barra Seca, Jaguaré, in the vicinity of the SBR (Inês Pinto, personal communication). The eagle's skin was prepared through artistic taxidermy, with its wings spread, and remained in the Pinto family's possession for several years before disappearing.

**FIGURE 4:**
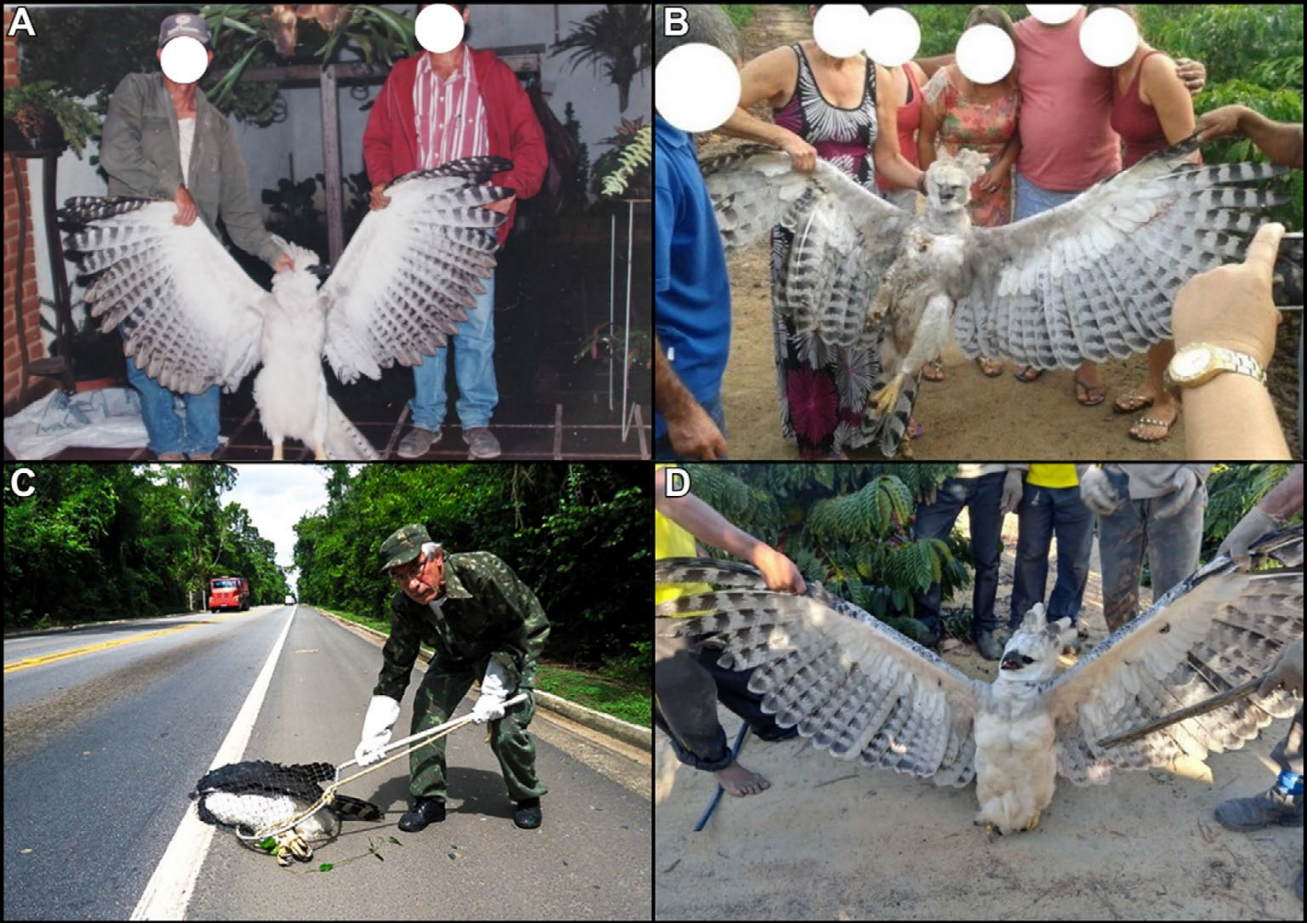
Harpy Eagles found dead in the VNR and SBR forest complex. (A) Juvenile female electrocuted in 1997 – I02; (B) Juvenile female electrocuted in 2014 – I03; (C) Adult female Harpy Eagle found roadkilled on BR‐101 in 2015 – I04 (Photo by Valdir Santos); (D) Juvenile female rescued by residents in 2021, later killed after being released – I05.

I02 and I03 were juvenile females recorded in 1997 (Aguiar‐Silva et al. 2012) and 2014, respectively, both found by members of the local community (Figure 4A,B, respectively). The carcass of I02 is housed at the Lorenzutti Museum in Linhares, ES (ML0120), and I03 is preserved at the Museum of Life Sciences (MCV151).

I04 was an adult female found roadkilled on April 8, 2015, at kilometer 103 of the BR‐101 highway, which crosses the SBR, and was reported by reserve staff (Figure 4C). The bird was rescued by SBR staff and transported by the Atlantic Forest Harpy Eagle Project team to the Veterinary Hospital of Vila Velha University, in Vila Velha, ES. However, it died the following day (Banhos et al. 2015). Examinations performed at the hospital revealed two lead bullets in the body, one chronic and one recent, along with bruises consistent with collision trauma (Banhos et al. 2015). The carcass was deposited at the Museum of Life Sciences (MCV150).

I05 was a juvenile female found alive on June 29, 2021 (Figure 4D), after falling in a coffee plantation on a rural property near the reserves. The eagle was initially captured by residents and confined in a cage. During the capture, one of the residents was injured by the bird's talons. The eagle was handed over to SBR staff and the Atlantic Forest Harpy Eagle Project team, who transported it for care within the reserve. It was monitored for ten days by Project biologists and veterinarians from the *Bicho Solto* *Saúde Animal* veterinary clinic, in São Mateus, ES, and showed no apparent health issues. After being cleared for release, the bird was returned to the wild on July 10, 2021, near the site where it was found, equipped with a radio transmitter, a standard band from the *Centro Nacional de Pesquisa e Conservação de Aves Silvestres* ‐ CEMAVE (number Z01023), and a microchip in the tarsus (number 95300001021136). However, on July 24, 2021, the eagle was found dead, most likely the victim of hunting, as the carcass presented an apparent gunshot wound. The specimen was deposited at the Museum of Life Sciences of the Federal University of Espírito Santo, in Vitória, ES (VAV0022).

## 4 Discussion

Occurrence records are essential for understanding a species' range and distribution. In the case of the Harpy Eagle, such records have facilitated the location and monitoring of nests, contributing to knowledge about the species in the Atlantic Forest.

Several studies based on biological samples collected within and around the SBR and VNR, many by local community members, and on nest monitoring have deepened our understanding of the Harpy Eagle ecology and conservation challenges. For example, genetic analyses of these samples revealed a decline in genetic diversity, likely resulting from ongoing habitat loss, fragmentation, and population reduction in the biome (Banhos et al. 2016). These samples also allowed for the examination of sex ratios across life stages, revealing a consistent female‐biased pattern among eaglets, juveniles, and adults in the population (Banhos et al. 2023). Furthermore, nest monitoring provided valuable insights into the species' feeding ecology, indicating a diverse diet that includes several threatened mammals such as the crested capuchin ( *Sapajus robustus* ), red howler monkey ( *Alouatta guariba clamitans* ), masked titi monkey ( *Callicebus personatus* ), and thin‐spined porcupine ( *Chaetomys subspinosus* ) (Kaizer et al. 2023). Together, these findings underscore the need for integrated conservation strategies that address both genetic and ecological dimensions of Harpy Eagle populations in the Atlantic Forest.

Although the earliest record analyzed dates to the 1970s, when a Harpy Eagle was poached near the SBR, and the species was also reported in the SBR Management Plan in 1981 (Instituto Brasileiro de Desenvolvimento Florestal ‐ IBDF 1981), as well as a 1985 report describing a Harpy Eagle found grounded on a road within the VNR, which was released after hydration (Peixoto and Peixoto 1986; Pacheco et al. 2003), there are even older records in the surrounding area. These include a specimen collected in 1929 near Juparanã Lagoon (AMNH 317240), in Linhares, approximately 5 km from the current reserves, and a nest recorded in 1944, in a *C. legalis* tree (Ruschi 1979), also in Linhares (Appendix S2). Recent (Appendix S1) and historical records (Appendix S2) highlight the long‐standing presence of Harpy Eagles in the region, reflecting the evolution of scientific knowledge, conservation efforts, and socio‐environmental conflicts in the territory.

Despite a conservation history dating back to the first half of the 20th century (Instituto Brasileiro de Desenvolvimento Florestal ‐ IBDF 1981; Instituto Chico Mendes de Conservação da Biodiversidade – ICMBio 2014; Rolim et al. 2016) the surrounding landscape has undergone extensive transformation due to population growth and agricultural expansion, processes largely accelerated by the construction of BR‐101 in the 1970s, a major federal highway with heavy traffic. The name ‘Sooretama’, meaning ‘land of animals’ in the Indigenous Tupi language, was originally given to one of the reserves in the 1940s and, reflecting the region's demographic expansion, was later adopted in 1994 as the name of a newly established municipality (Câmara Municipal de Sooretama 2025).

According to MapBiomas (MapBiomas 2026), forest formation cover declined by 12,232 ha (10%) between 1985 and 2024 in the municipalities of Sooretama (2287 ha, 7.78%), Linhares (4028 ha, 5.23%), Jaguaré (4223 ha, 28.37%), and Vila Valério (1692 ha, 21.07%), which surround the SBR and VNR. An additional decline of 13,791 ha (4.67%) was recorded in other municipalities in ES with Harpy Eagle records. These changes have resulted in significant habitat loss and increased wildlife mortality.

Although there are recent records of Harpy Eagles in the Atlantic Forest of the mountainous region of ES (Raton 2012; Magnago 2018; Ramaldes 2026) (Appendix S2), nests are known only from Tabuleiro Forest, in northern ES and southern BA (Kaizer et al. 2023; Luz et al. 2005; Aguiar‐Silva et al. 2012). Within this region, the forests of southern BA also exhibit a similarly significant history of Harpy Eagle nesting and human–Harpy conflict as observed in northern ES, between 300 km and 470 km away. This suggests the findings of this study may also be relevant there. Older nesting records have also been reported in the southern portion of the Atlantic Forest, particularly in Misiones, Argentina (Chebez et al. 1989; de Lucca 1996; Anfuso et al. 2008), and a recent record of a juvenile in the Turvo State Park, Rio Grande do Sul, in Brazil (Meller and Guadagnin 2016). However, new nesting records are needed to better understand the species' current status and distribution in the region.

### 4.1 Community Records

Most records were obtained in the VNR (*n* = 56), a privately protected area owned by the Vale company, which has its own access and surveillance policies (Rolim et al. 2016). This reserve is not part of the National System of Conservation Units (SNUC) (Brasil 2000), the legislation governing the management of officially recognized protected areas in Brazil. Despite this, the company promotes community participation within the VNR, encouraging recreational tourism, nature appreciation, and environmental education.

In contrast, the SBR, with 26 records, is a federal public conservation unit under full protection, classified as a Biological Reserve and part of the SNUC (Brasil 2000), where public use is restricted to scientific research and environmental education (Instituto Chico Mendes de Conservação da Biodiversidade – ICMBio 2014). Furthermore, the SBR has fewer access roads compared to the VNR (Figure 2), which limits the movement of people and may explain the disparity in the number of records between the two areas. No records were obtained in the Mutum Preto and Recanto das Antas PNHRs, the small ones in the region. These reserves are maintained by the Suzano company and are part of the SNUC (Brasil 2000), but lack infrastructure for public visitation.

In fact, the recorded locations were strongly associated with the road network crossing the reserves, indicating a bias related to human access. We observed that most records (94.19%) were made inside the reserves, with only five records (5.81%) in the surrounding areas (Figure 2). Although the surroundings of the reserves are more accessible and more frequently used by local people, only a few external records were reported, mostly involving animals under threat. This may suggest a lower perception of the species among local people, although no systematic effort was made to gather information on the Harpy Eagle from communities in the buffer zones.

In contrast, a large portion of the records obtained inside the reserves came from workers and birdwatchers. Most of these observations were accompanied by high‐quality photographs submitted to the Brazilian digital platform WikiAves (https://www.wikiaves.com.br/), which enabled the identification of age class of the individuals in several cases. As of March 2026, the platform contained 95 images and audio recordings of Harpy Eagles in the VNR and SBR, of which 12 correspond to records compiled in this study (Betkowski 2015; Magnago 2015a; Magnago 2015b; Barros 2016; Briso 2017; Bonfa 2017; Bonfa 2024a; Bonfa 2024b; Bonfa 2024c; Bonfa 2024d; Bonfa 2025; Rennó 2009) (Appendix S1). The remaining images and audio recordings were associated with the same records or were obtained by workers and birdwatchers visiting known nests. This contribution highlights the potential of citizen science and the importance of community engagement in generating knowledge about Harpy Eagle ecology and conservation (Curti and Valdez 2009), as well as the species potential as a tourism attraction (Miranda et al. 2022) in the region.

The monthly pattern of Harpy Eagle records within the reserves may be linked to their breeding season, which occurs between September and March, corresponding to the rainy season in Atlantic Forest (Develey and Peres 2000). During this period, pairs tend to stay closer to their nests, focusing on egg incubation and eaglet protection, which may hinder sightings of individuals away from the nest. Therefore, the dry season in the Atlantic Forest appears to be a more favorable time for incidental Harpy Eagle observations within the reserves.

### 4.2 Nest Discovery

The first nest was found in 1992 by a researcher and became known to VNR staff and visitors for years (Pacheco et al. 2003; Aguiar‐Silva et al. 2012). Later, observations by researchers and staff led to the discovery of a new nest in 2010 (Aguiar‐Silva et al. 2012). In 2016, another nest was located during a birdwatching walk, highlighting the role of citizen science in monitoring the species in the region.

Recent community reports have also played a key role, leading to the discovery of five additional nests by the Atlantic Forest Harpy Eagle Project. Information gathered from the community guided the search efforts, as the presence of pairs and juveniles often indicates proximity to a nest, and this strategy has proven effective. Most nests (*n* = 4) were found using drones, underscoring the value of this technology for biodiversity monitoring (Barros et al. 2019; Pedro et al. 2024).

Importantly, the trees used by Harpy Eagles for nesting in the region average 39.25 m in height, which is taller than the mean forest canopy height in the Tabuleiro Atlantic Forest, where the upper arboreal stratum is discontinuous, with trees ranging from 19 to 31 m in height (mean = 24 m) and the canopy reaching up to 40 m (Peixoto et al. 1995). At least four different species were used within the reserves, including some dead trees and species that are threatened with extinction. Ruschi (Ruschi 1979) also documented tree species used by Harpy Eagles in two historical records from ES: one *Lecythis urnigera* Cambess, recorded before 1946, and one *C. legalis* in 1944 (Appendix S2). Thus, at least five species have been used by Harpy Eagles in the region. Of these, three were already known from previous studies in the Atlantic Forest: *C. legalis* (Miranda et al. 2020; Ruschi 1979; Novaes et al. 2010), *A. concinnum* (Miranda et al. 2020; Novaes et al. 2010), and *L. urnigera* (Ruschi 1979), while *E. macrophylla* and *M. longifolia* are newly reported here. These findings highlight the importance of investigating nest site selection criteria and the environmental impacts affecting the availability of these key reproductive resources in the Atlantic Forest.

Among the six nests recorded in the VNR, two likely belonged to the same pair, as they were relatively close—approximately 0.96 km apart—and one had collapsed (N03) before the other was discovered (N07). In the SBR, the three nests (N04, N06 and N09) also likely belonged to the same pair, given their proximity (N06 and N09 about 1.43 km and 1.33 km from the N04, respectively; Figure 2), the death of the tree that supported the earlier nest, and the fact that the three nests were not used simultaneously. A study in Panama found nests used by the same pair spaced an average of 1.1 km apart (Vargas and Vargas 2011).

Based on the nests found, we infer that the VNR currently harbors at least four breeding pairs of Harpy Eagles, of which three can still be observed at their nests and are being monitored. However, the pair associated with the nest found in 2010 may be using an alternative nest, as breeding attempts with egg‐laying were recorded only in 2018–2019, unsuccessful. In contrast, the SBR, with only three nests attributed to a single pair, requires greater search effort for new nests, as it is larger than the VNR and may host additional Harpy Eagle families.

It is worth highlighting that the VNR and SBR forests are connected and should maintain a continuous population of the species. Considering that a Harpy Eagle pair may occupy a home range of 10–79 km^2^ around its nest, as observed in Panama and Venezuela, respectively (Vargas et al. 2006), the 53,000 ha of forest that make up the complex formed by the SBR, VNR, and adjacent reserves could theoretically support between seven and 53 breeding pairs. However, given the ecological resource limitations (emergent trees and prey) and local threats in the Atlantic Forest, it is likely that the higher end of this estimate is far from being reached. The home range of a Harpy Eagle pair under the specific conditions of the Atlantic Forest is also a relevant question to be investigated, as it can contribute to understanding the species' population size within the biome and the amount of area required for its conservation.

### 4.3 Local Threats to Harpy Eagles

Habitat loss is considered one of the main threats to the Harpy Eagle throughout its range (BirdLife International 2022), as it is a strictly forest‐dependent species. In the Central Atlantic Forest Corridor, SBR‐VNR represents the largest remaining area with confirmed presence of the species. However, outside these protected areas, deforestation persists in the surrounding landscape, reducing connectivity among the remaining forest fragments, isolating populations, restricting gene flow, and increasing the risk of local extinctions (Banhos et al. 2016).

The presence of live juveniles and active nests within the reserves underscores the importance of the area for Harpy Eagle reproduction. This region may function as a source population within a broader metapopulation matrix, potentially contributing to the recolonization of areas where the species has been locally extirpated in the Atlantic Forest. However, only three eaglets were observed in nests, despite multiple breeding attempts by adult pairs, suggesting low reproductive success in the region. Additionally, some of the recruited juveniles were killed in the surrounding areas. Low reproductive rates, poor breeding success, and high juvenile mortality together increase the risk of extinction for Harpy Eagles in this region.

Records of Harpy Eagles killed by electrocution, poaching, and road collisions highlight the significant challenges the species faces in the region. Electrocution of the Harpy Eagle has also been reported in deforested areas of the Amazon (Gusmão et al. 2020). As one of the leading causes of bird mortality worldwide (Lehman et al. 2007; Guil and Pérez‐García 2022), electrocution underscores the urgent need for adaptations and preventive measures to mitigate the risks associated with power infrastructure near the reserves.

The longstanding and persistent persecution of Harpy Eagles within and around the reserves remains a cause for concern. The case of a juvenile Harpy Eagle shot and killed after being rescued and released underscores the vulnerability of these birds, even following management interventions. Behaviors such as perching in open areas, combined with limited local knowledge about the species, contribute to its persecution and poaching across its range (de Freitas et al. 2014; Trinca et al. 2008; Giraldo‐Amaya et al. 2021; Miranda, Peres, and Downs 2021; Banhos et al. 2015). Poaching is a persistent and historically documented threat in both the VNR and SBR, affecting multiple species (Chiarello 2000; Ferreguetti et al. 2019; Ferreguetti et al. 2018; Fontes et al. 2020). The ongoing conflict between Harpy Eagles and local communities highlights the urgent need for educational campaigns to raise public awareness about the species' ecological importance, rarity, as well as the consequences of hunting protected wildlife.

The roadkill of an adult female Harpy Eagle, combined with evidence of gunshot wounds prior to the collision (Banhos et al. 2015), illustrates the multiplicity of threats faced by each individual. The BR‐101 highway, which cuts through the forest complex, represents a major risk factor, as numerous animal species have been roadkilled in the region, including jaguars ( *Panthera onca* ) (Srbek‐Araujo et al. 2015), pumas ( *Puma concolor* ) (Srbek‐Araujo et al. 2015), lowland tapirs ( *Tapirus terrestris* ) (Banhos et al. 2021), and even volant animals such as bats (Damásio et al. 2021) and birds. Reducing vehicle speeds in the area and more monitoring and control systems may be effective in reducing such collisions (Srbek‐Araujo et al. 2015; Banhos et al. 2021; Damásio et al. 2021), especially for species that may fly over the road at the same height as passing vehicles.

The proximity of nests to human‐modified areas (Figure 2), ranging from 1 to 3.5 km from highway and 250 m–5 km from power lines and rural communities, is also a cause for concern. Records of individuals outside reserve boundaries suggest that Harpy Eagles use the surrounding landscape as part of their home range or during dispersal. The juvenile dispersal period is particularly critical, as individuals leaving their natal territories become more vulnerable to anthropogenic threats. Although the areas surrounding the reserves are largely dominated by agriculture, they still contain numerous forest fragments (Figure 2), which may be used by both Harpy Eagles and their prey.

One of the nests in the VNR (N08) is located outside the buffer zone (*Zona de Amortecimento* ‐ [ZA]) of the SBR (Figure 2). The buffer zone provides enhanced legal protection to the surrounding territory. Therefore, expanding the SBR ZA to encompass all areas with Harpy Eagle records—along with formally establishing a conservation unit in the VNR region—would help strengthen the protection of the region's rich biodiversity and contribute to the SNUC (Brasil 2000) and Sustainable Development Goal 15—Life on Land (United Nations ‐ UN 2015).

The Hileia Baiana forest represents a landscape where both the Harpy Eagle and the species it interacts with face severe extinction risks, including many of its prey species (Kaizer et al. 2023) and most of the large trees used for nesting, which have become ecologically scarce. In this context, the conservation of the Harpy Eagle must consider the broader ecosystem in which it occurs. Conversely, as an umbrella species, conservation actions targeting Harpy Eagles can strategically benefit other species it interacts with, both flora (such as tree species used for nesting) and fauna (especially prey), fostering integrated biodiversity conservation.

Ensuring the long‐term viability of one of the last reproductive populations of Harpy Eagles in the Atlantic Forest requires strategies that promote coexistence with local communities. Mitigating the identified threats through public policies, infrastructure adjustments, environmental education, and partnerships with local residents is essential for effective conservation.

## 5 Conclusions

1. This study highlighted the crucial role of the local community and citizen science in generating data on Harpy Eagles in one of the species' last reproductive refuges in the Atlantic Forest. Records from tourists, workers, and local residents, combined with researchers' efforts, were instrumental in locating nests and expanding knowledge about the species.
2. It is important to note that there has been a growing effort in observation and research, combined with greater logistical and technological access to the narrow edges of the Atlantic Forest. Thus, the apparent increase in records may reflect intensified detection effort and improved knowledge of the species, rather than necessarily favorable population conditions.
3. Most records were obtained within the reserves, near areas accessed by engaged visitors. However, the surrounding landscape proved critical for the dispersal and survival of both juveniles and adults due to threats such as hunting, electrocution, and roadkill.
4. The recorded conflicts reveal a concerning scenario, given the small and isolated remaining population, underscoring the urgent need to strengthen protection mechanisms in the area.
5. The conservation of Harpy Eagles in their last reproductive refuges depends on the engagement of surrounding communities, scientific research, and the implementation of effective public policies.

## Author Contributions

**Brener Fabres:** conceptualization (equal), data curation (equal), formal analysis (equal), investigation (equal), methodology (equal), project administration (equal), visualization (equal), writing – original draft (equal). **Tânia Margarete Sanaiotti:** conceptualization (equal), data curation (equal), formal analysis (equal), funding acquisition (supporting), investigation (equal), methodology (equal), project administration (equal), supervision (supporting), visualization (equal), writing – original draft (equal). **Gustavo Magnago:** data curation (equal), formal analysis (equal), funding acquisition (supporting), investigation (supporting), methodology (equal), visualization (supporting). **Gabriel Scaldaferro Bonfa:** investigation (supporting), methodology (supporting), visualization (equal). **Henrique Mariano Martins:** data curation (supporting), investigation (supporting), methodology (supporting), visualization (equal). **Paulo Quadros de Menezes:** data curation (supporting), formal analysis (supporting), investigation (supporting), methodology (supporting), visualization (equal), writing – original draft (supporting). **José Alves da Costa Filho:** formal analysis (equal), investigation (supporting), methodology (supporting), visualization (equal). **Mylena Kaizer:** formal analysis (supporting), investigation (supporting), methodology (supporting), visualization (equal). **Carlos Hartur Ribeiro Nóia:** formal analysis (supporting), investigation (supporting), methodology (supporting), visualization (equal). **Frederico Pereira de Castro Andrade:** investigation (supporting), methodology (supporting), visualization (equal). **Francisca Helena Aguiar‐Silva:** data curation (supporting), formal analysis (supporting), investigation (supporting), methodology (supporting), visualization (equal). **Olivier Jaudoin:** investigation (supporting), methodology (supporting), visualization (supporting). **Ana Carolina Srbek‐Araujo:** data curation (supporting), investigation (supporting), methodology (supporting), visualization (equal). **Geovane Souza Siqueira:** investigation (supporting), methodology (supporting), visualization (equal). **Aureo Banhos:** conceptualization (equal), formal analysis (equal), funding acquisition (lead), investigation (equal), methodology (equal), project administration (equal), resources (equal), supervision (lead), visualization (equal), writing – original draft (equal).

## Funding

This work was supported by Vale S.A., the Espírito Santo Research and Innovation Support Foundation (FAPES), the Brazilian National Council for Scientific and Technological Development (CNPq), the Beauval Nature Association, and the Brazilian Fund for Biodiversity (FUNBIO).

## Consent

All authors consent to their participation in this study. The authors consent to the publication of this study by Scientific Reports.

## Conflicts of Interest

The authors declare no conflicts of interest.

## Supporting information


**Appendix S1:** Records of Harpy Eagles in the Sooretama Biological Reserve (SBR) and Vale Natural Reserve (VNR) from early 1970s to 2026.

**Appendix S2:** Municipalities and reserves in the state of Espírito Santo, Brazil, with Harpy Eagle records and their proximity to the Sooretama Biological Reserve (SBR) and Vale Natural Reserve (VNR), from 1929 to 2026.

## Data Availability

All data supporting the findings of this study are fully available within the main text of the article and in the appendices. No additional datasets were generated or analyzed.
