# Supplementary material for: Local Knowledge and Human–Wildlife Conflict in the Conservation of the Harpy Eagle ( Harpia harpyja ) in Its Main Refuge in the Atlantic Forest
Source: Ecol Evol. 2026 May 12;16(5):e73499. doi: 10.1002/ece3.73499 (PMC13161988; doi:10.1002/ece3.73499)
Supplement: Supplementary file 1 — Appendix S1: Records of Harpy Eagles in the Sooretama Biological Reserve (SBR) and Vale Natural Reserve (VNR) from early 1970s to 2026. [file ECE3-16-e73499-s001.docx]

**Supplementary Table 1. Records of Harpy Eagles in the Sooretama Biological Reserve (SBR) and Vale Natural Reserve (VNR) from last 1970 to 2026.**

| **Record** | **Date** | **Locality** | **Place** | **Record Type** | **Life Stage** | **Author** | **Source** |
| --- | --- | --- | --- | --- | --- | --- | --- |
| 1 | Late 1970s | Barra Seca/Jaguaré |  | Dead specimen/ Photo | Adult | Antônio Pinto and Inês Pinto - local | Fabres et al. (this study) |
| 2 | 1981 | SBR |  | Report |  | Instituto Brasileiro de Desenvolvimento Florestal - researchers | Instituto Brasileiro de Desenvolvimento Florestal 1981 |
| 3 | 01/1985 | VNR | Gavião-real road/Macanaíba-pele-de-sapo road | Rescue/Release/  Photo | Adult | A. L. Peixoto and O. L. Peixoto - researchers | Peixoto & Peixoto 1986; Pacheco et al. 2003 |
| 4 | 08/1992 | VNR |  | Sighting |  | Galetti et al. - researchers | Galetti et al. 1997 |
| 5 | 12/1992 | VNR |  | Sighting |  | Galetti et al. - researchers | Galetti et al. 1997 |
| 6 | 1992 | VNR | Macanaíba-pele-de-sapo road | Nest | Eaglet in the nest | Bret M. Whitney - researcher | Galetti et al. 1997; Pacheco et al. 2003; Aguiar-Silva et al. 2012 |
| 7 | 1995-1996 | VNR | Gavião-real road | Sighting |  | E. Costa – VNR staff member | Aguiar-Silva et al. 2012 |
| 8 | 1996 | SBR | Córrego do Cupido | Sighting |  | SBR staff members | Pacheco et al. 2003 |
| 9 | 1997 | Santa Terezinha Farm/Linhares | Rancho Alto, Santa Terezinha farm, Caliman Agrícola | Electrocution/  Dead specimen/Photo | Juvenile female | João Antônio Rocha - local | ML0353; Aguiar-Silva et al. 2012 |
| 10 | 2000 | VNR | Onça-pintada road/Jequitibá-rosa road | Sighting |  | P. R. Paz - VNR staff member | Aguiar-Silva et al. 2012 |
| 11 | 2001 | VNR | Onça-pintada road/Caingá road | Sighting | Adult | J. C. Silva – VNR staff member | Aguiar-Silva et al. 2012 |
| 12 | 2002 | VNR | Onça-pintada road | Sighting |  | A. C. Santos – VNR staff member | Pacheco et al. 2003 |
| 13 | 2002 | SBR | SBR headquarters | Sighting | Adult | Guanadir Gonçalves – SBR staff member | Fabres et al. (this study) |
| 14 | 2003 | VNR | Gavião-real road | Sighting | Adult | J. C. Silva – VNR staff member | Aguiar-Silva et al. 2012 |
| 15 | 2004 | VNR | Gavião-real road/Roxinho road | Sighting | Adult | J. C. Silva, A. Hartuique and E. Silva – VNR staff members | Aguiar-Silva et al. 2012 |
| 16 | 2005 | SBR | Km 105.5 of BR-101 | Sighting |  | Adeildon Hartuique – VNR staff member | Fabres et al. (this study) |
| 17 | 2005 | VNR | Onça-pintada road/Gavião-real road | Sighting |  | D. Folli and M. Cruz – VNR staff members | Aguiar-Silva et al. 2012 |
| 18 | 08/2005 | VNR | Oiticica road | Photo | Adult | Ana Carolina Srbek – VNR staff member | Srbek-Araujo & Chiarello 2026 |
| 19 | 2005-2006 | VNR | Onça-pintada road/Ipê Amarelo road | Sighting |  | E. Costa - VNR staff member | Aguiar-Silva et al. 2012 |
| 20 | 2005-2006 | VNR | Oiticica road | Sighting | Adult | E. Costa - VNR staff member | Aguiar-Silva et al.2012 |
| 21 | 2006 | SBR | Jequitibá-rosa tree/Environmental Education trail | Feather | Adult female^61^ | Eliton Lima and Valdir Martins Santos - SBR staff members | Fabres et al. (this study) |
| 22 | 2007-2008 | VNR | SBR/VNR firebreak | Sighting | Juvenile | J. C. Gonçalves and P. P. Reis – VNR staff members | Aguiar-Silva et al. 2012 |
| 23 | 03/2009 | VNR | Onça-pintada road | Photo | Adult | A. Hartuique, J. S. Santos, A. S. Coutinho, Alan and Ectori - VNR staff members | Aguiar-Silva et al. 2012 |
| 24 | 04/07/2009 | VNR | SBR/VNR firebreak | Sighting/Audio record | Adult | Ana Carolina Srbek – VNR staff member; Bruno Rennó and Rafael Bessa - researchers | Aguiar-Silva et al. 2012;  Rennó 2009 |
| 25 | 2009 | Santa Teresinha Farm/Linhares | Santa Teresinha farm | Sighting | Adult pair | J. Negrelli – SBR staff member | Aguiar-Silva et al. 2012 |
| 26 | 2009 | VNR | Boleira road | Sighting |  | Geovane Siqueira – VNR staff member | Aguiar-Silva et al. 2012 |
| 27 | 2010 | SBR | Jequitibá-rosa tree/Environmental Education trail | Vocalization |  | Valdir Martins Santos – SBR staff member | Fabres et al. (this study) |
| 28 | 02/09/2010 | VNR | Oiticica road | Sighting | Adult pair | F. Helena Aguiar-Silva, Olivier Jaudoin - Harpy Eagle Project – researchers; Ana Carolina Srbek – VNR staff member | Aguiar-Silva et al. 2012 |
| 29 | 02/09/2010 | VNR | Oiticica road | Nest | Adult in the nest | F. Helena Aguiar-Silva, Olivier Jaudoin - Harpy Eagle Project – researchers; Ana Carolina Srbek – VNR staff members | Aguiar-Silva et al. 2012 |
| 30 | 09/2010 | SBR | Km 103.6 of BR-101 | Sighting |  | Ruan Preato Deolindo and Luciano Medeiros - SBR staff members | Fabres et al. (this study) |
| 31 | 07/02/2011 | VNR | Onça-pintada road | Sighting/Feather | Adult male | D. Folli, C. C. Silva and A. A. Cruz -– VNR staff members | Aguiar-Silva et al. 2012 |
| 32 | 29/06/2011 | VNR | SBR/VNR firebreak | Feather | Adult female | SBR staff members | Aguiar-Silva et al.2012 |
| 33 | 08/2011 | VNR | Roxinho road | Sighting | Adult | Geovani Siqueira - VNR staff member | Aguiar-Silva et al. 2012 |
| 34 | 04/06/2012 | VNR | Pequi-vinagreiro trail | Feather | Adult  male | K. T. Biancardi – VNR staff member | Aguiar-Silva et al.2012 |
| 35 | 02/05/2013 | SBR | Pond of Macuco | Sighting | Adult | Leonardo Merçon - photographer and researchers | Fabres et al. (this study) |
| 36 | 09/07/2013 | VNR | Zamboa road | Sighting | Adult | Ana Carolina Srbek and José Simplício dos Santos -VNR staff members | Fabres et al. (this study) |
| 37 | 29/08/2013 | VNR | Milanesi firebreak | Feather | Adult | Luiza Avelar – VNR staff member | Fabres et al. (this study) |
| 38 | 08/2013 | VNR | Gavião-real road | Vocalization |  | José Nilton da Silva - Harpy Eagle Project researcher | Fabres et al. (this study) |
| 39 | 21/11/2013 | VNR | SBR/VNR firebreak | Vocalization | Adult pair | Ana Carolina Srbek and José Simplício dos Santos – VNR staff members | Fabres et al. (this study) |
| 40 | 15/01/2014 | VNR | Gavião-real road/Mantegueira road | Photo | Juvenile | Luiza Avelar and Cecilia Kierulff – VNR staff members | Fabres et al. (this study) |
| 41 | 18/05/2014 | Córrego Farias District/Linhares | Santa Rita Farm, Córrego Farias District | Electrocution/  Dead specimen/Photo | Juvenile female | José Antônio Gumieiro and Layane Tose - locals | MCV151; Fabres et al. (this study) |
| 42 | 15/09/2014 | SBR | C3 Research trail 800 m west from km 104 of BR-101 | Vocalization |  | José Nilton da Silva - Harpy Eagle Project researcher | Fabres et al. (this study) |
| 43 | 24/09/2014 | SBR | Tesouro road | Vocalization |  | José Nilton da Silva - Harpy Eagle Project researcher | Fabres et al. (this study) |
| 44 | 09/02/2015 | VNR | Peroba-osso road | Photo/ Audio record | Adult | Gustavo Magnago and Leticia Belgi Bissoli Magnago - birdwatchers | Magnago G.2015 |
| 45 | 08/04/2015  09/04/2015 | SBR | Km 103 of BR-101 | Road-killed/ Rescue/  Dead specimen/Photo | Adult female | Valdir Martins Santos – SBR staff member | MCV150; Banhos *et al.*^21^ |
| 46 | 05/2015 | SBR | Do Meio road/ES-368 | Feather | Adult female | Carolina Demétrio Ferreira – UFES researcher | Fabres et al. (this study) |
| 47 | 02/11/2015 | VNR | Onça-pintada road | Photo | Juvenile | Justiniano Magnago and Eduardo Patrial - birdwatchers | Magnago J. 2015 |
| 48 | 10/12/2015 | SBR | RAPELD O3 trail, 2500 m from km 104 BR-101 | Photo/Audio record | Adult pair | Marcelo Barreiros and Samuel Betkowski – Concremat researchers | Betkowski 2015 |
| 49 | 02/2016 | VNR | Peroba-osso road | Vocalization |  | Justiniano Magnago - birdwatcher | Fabres et al. (this study) |
| 50 | 28/03/2016 | SBR | Mata Azul, SBR/VNR firebreak | Feather | Adult male | Roni Rodrigo – SBR staff member | Fabres et al. (this study) |
| 51 | 27/08/2016 | VNR | Peroba-osso road | Nest | Adult in the nest | Justiniano Magnago, João Sergio Barros and Angelo Frederico - birdwatchers | Barros 2016 |
| 52 | 12/2016 | SBR | Km 104 of the highway BR-101 | Sighting | Adult | Brener Fabres – local | Fabres et al. (this study) |
| 53 | 20/03/2017 | Córrego Farias District/Linhares | Córrego Farias District road | Photo | Adult | Gustavo Magnago and André Luiz Briso - birdwatchers | Briso 2017 |
| 54 | 07/05/2017 | VNR | Aderne road | Feather | Adult female | David Costa Braga and Sueli Huber Oliveira - Felinos Project researchers | Fabres et al. (this study) |
| 55 | 13/05/2017 | VNR | Onça-pintada road /Bomba-d’água road | Sighting/Audio record | Adult | Gabriel Bonfá - birdwatcher | Bonfa 2017 |
| 56 | 08/08/2017 | SBR | RAPELD O3 trail, 2500 m from km 105 BR-101 | Nest | Eaglet in the nest | Aureo Banhos, Frederico Pereira de Andrade and Kézia Catein - Atlantic Forest Harpy Eagle Project researchers | Fabres et al. (this study) |
| 57 | 01/03/2018 | VNR | Aderne road | Sighting |  | Hilton Entringer Júnior - Felinos Project researcher | Fabres et al. (this study) |
| 58 | 01/03/2018 | VNR | SBR/VNR firebreak | Feather | Juvenile female | Hilton Entringer Júnior - Felinos Project researcher | Fabres et al. (this study) |
| 59 | 29/01/2019 | VNR | Carneiro road | Sighting | Adult pair | Gustavo Magnago - Atlantic Forest Harpy Eagle Project researcher | Fabres et al. (this study) |
| 60 | 19/07/2019 | VNR | Onça-pintada road | Nest | Vacant nest | Gustavo Magnago, Gabriel Bonfá & Brener Fabres - Atlantic Forest Harpy Eagle Project researchers | Fabres et al. (this study) |
| 61 | 25/09/2019 | SBR | RAPELD L2 trail, 2500 m from km 104 BR-101 | Feather | Adult female | Tomas de Lima Rocha, Amanda Del'Maestro and Tainara de Alencar - Herpeto Capixaba Project researchers | Fabres et al. (this study) |
| 62 | 21/03/2021 | SBR | RAPELD O3 trail, 1000 m from km 105 BR-101 | Nest | Vacant nest | Carlos Hartur Ribeiro Noia - Atlantic Forest Harpy Eagle Project researcher | Fabres et al. (this study) |
| 63 | 05/2021 | VNR | Near the Jueirana-vermelha road | Photo | Adult | VNR staff members | Fabres et al. (this study) |
| 64 | 29/06/2021  10/07/2021  24/07/2021 | Barra Seca/Jaguaré | Near the "mata azul" | Rescue/  Release/  Dead specimen/ Photo | Juvenile female | Robson Luiz Giuzolfe and Silvado Lagas - locals; Marcel Redling Moreno and Cristiane Aguiar – SBR staff members; Aureo Banhos, Brener Fabres, Olivier Jaudoin, Dante Meller, Jailson Souza and Carlos Hartur Ribeiro Noia - Atlantic Forest Harpy Eagle Project researchers | VAV0022; Fabres et al. (this study) |
| 65 | 09/2021 | SBR | Km 106 of the BR-101 | Sighting | Juvenile | Damiane Paolo Rocha - local | Fabres et al. (this study) |
| 66 | 01/2022 | VNR | Firebreak/Peroba-amarela road | Nest | Adult in the nest | Carlos Hartur Ribeiro Noia - Atlantic Forest Harpy Eagle Project researcher | Fabres et al. (this study) |
| 67 | 09/02/2022 | SBR | RAPELD L2 trail, 1900 m from km 104 BR-101 | Photo | Adult | Canopy camera trap — Atlantic Forest Harpy Eagle Project researchers | Fabres et al. (this study) |
| 68 | 19/06/2022 | VNR | Onça-pintada road | Sighting | Adult pair | Henrique Mariano, Jailson Souza and João Vitor Pasinato - Atlantic Forest Harpy Eagle Project researchers | Fabres et al. (this study) |
| 69 | 25/06/2022 | VNR | Boleira road | Nest | Adult in the nest | Aureo Banhos, Letícia Guerra and Henrique Mariano - Atlantic Forest Harpy Eagle Project researchers | Fabres et al. (this study) |
| 70 | 11/07/2022 | SBR | Km 102 of the BR-101 | Sighting | Adult | Henrique Mariano - Atlantic Forest Harpy Eagle Project researcher | Fabres et al. (this study) |
| 71 | 01/09/2022 | SBR | Ring road, 1500 m from km 102 BR-101 | Photo | Adult | SBR - staff members | Fabres et al. (this study) |
| 72 | 14/11/2022 | VNR | Gavião-real road | Sighting | Adult | Brener Fabres - Atlantic Forest Harpy Eagle Project researcher | Fabres et al. (this study) |
| 73 | 15/07/2023 | VNR | Onça-pintada road | Photo | Adult | Brener Fabres - Atlantic Forest Harpy Eagle Project researcher | Fabres et al. (this study) |
| 74 | 15/12/2023 | SBR | Do Meio road, ES-368 | Vocalization | Adult | Henrique Mariano - Atlantic Forest Harpy Eagle Project researcher | Fabres et al. (this study) |
| 75 | 11/2023 | SBR | Km 104 of BR-101 | Sighting | Adult | Romildo Ferreira da Silva - local | Fabres et al. (this study) |
| 76 | 13/02/2024 | VNR | Peroba-osso road | Photo | Adult | Gabriel Bonfá - birdwatcher | Bonfa 2024a |
| 77 | 27/04/2024 | VNR | Peroba-osso road | Vocalization | Adult | Paulo Quadros, Mylena Kaizer, Marcelio Cunha and Henrique Mariano - Atlantic Forest Harpy Eagle Project researchers | Fabres et al. (this study) |
| 78 | 28/04/2024 | VNR | Onça-pintada road | Vocalization | Adult | Henrique Mariano - Atlantic Forest Harpy Eagle Project researcher | Fabres et al. (this study) |
| 79 | 28/04/2024 | VNR | Peroba-osso road | Photo/Audio record | Adult | Gabriel Bonfá - birdwatcher | Bonfa 2024b |
| 80 | 29/04/2024 | VNR | Carneiro road | Photo | Adult pair | Paulo Quadros, Marcelio Cunha and Henrique Mariano - Atlantic Forest Harpy Eagle Project researchers | Fabres et al. (this study) |
| 81 | 25/05/2024 | VNR | Carneiro road | Photo | Adult pair | Thassiane Targino, Marcélio Cunha and Henrique Mariano - Atlantic Forest Harpy Eagle Project researchers | Fabres et al. (this study) |
| 82 | 13/06/2024 | VNR | Birdwatching Tower/Accommodation road | Photo | Adult | Gabriel Bonfá - birdwatcher | Bonfa 2024c |
| 83 | 28/06/2024 | VNR | Peroba-osso road/Onça-pintada road | Photo | Adult | Gabriel Bonfá - birdwatcher | Bonfa 2024d |
| 84 | 09/03/2025 | VNR | Peroba-osso road | Photo | Adult | Gabriel Bonfá - birdwatcher | Bonfa 2025 |
| 85 | 08/08/2025 | VNR | Bomba D'Água road | Feather | Adult | Ana Carolina Srbek - Felinos Project researchers | Fabres et al. (this study) |
| 86 | 08/11/2025 | SBR | Mata azul | Feather | Adult | SBR staff members | Fabres et al. (this study) |
| 87 | 17/12/2025 | SBR | Abóbora road | Vocalization |  | Marcos Raposo - UFRJ researcher | Fabres et al. (this study) |
| 88 | 19/01/2026 | SBR | RAPELD O4 trail, 3500 m from km 106 BR-101 | Nest | Vacant nest | José Nilton da Silva and Lucas Damásio - Atlantic Forest Harpy Eagle Project researchers | Fabres et al. (this study) |
