# Supplementary material for: Local Knowledge and Human–Wildlife Conflict in the Conservation of the Harpy Eagle ( Harpia harpyja ) in Its Main Refuge in the Atlantic Forest
Source: Ecol Evol. 2026 May 12;16(5):e73499. doi: 10.1002/ece3.73499 (PMC13161988; doi:10.1002/ece3.73499)
Supplement: Supplementary file 2 — Appendix S2: Municipalities and reserves in the state of Espírito Santo, Brazil, with Harpy Eagle records and their proximity to the Sooretama Biological Reserve (SBR) and Vale Natural Reserve (VNR), from 1929 to 2026. [file ECE3-16-e73499-s002.docx]

| **Supplementary Table 2. Municipalities and reserves in the state of Espírito Santo, Brazil, with Harpy Eagle records and their proximity to the Sooretama Biological Reserve (SBR) and Vale Natural Reserve (VNR), from 1929 to March 2026.** | | | | |
| --- | --- | --- | --- | --- |
| **Year** | **Locality** | **Municipality** | **Information** | **Source** |
| 1929 | Juparanã Lagoon | Linhares | A specimen was collected at Juparanã Lagoon, Linhares municipality, in 1929, by Emil Kaempfer, and is housed at the American Museum of Natural History, New York (AMNH 317240). Juparanã Lagoon is approximately 5 km south of the current VNR and SBR. | AMNH 317240 |
| 1936–1951 | Professor Mello Leitão Biology Museum | Santa Teresa | Ruschi^37^ reported the species at the Professor Mello Leitão Biology Museum headquarters, in Santa Teresa municipality, between 1936 and 1951. The museum is located approximately 100 km southwest of the VNR and SBR. | Ruschi 1965 |
| 1937 |  | Colatina | Sick^47^ reported a nest in 1937 in Colatina municipality, approximately 60 km southwest of the VNR and SBR. | Sick 1997 |
| 1939-1942 | Caboclo Farm | Conceição da Barra | Ruschi reported the presence of a Harpy Eagle during a wildlife survey conducted between 1939 and 1942, during the clearing of 5,000 hectares of forest on Caboclo farm, in Conceição da Barra. The municipality is located approximately 60 km north of the VNR and SBR. | Ruschi 1954 |
| 1939-1976 | Santa Lúcia Ecological Station | Santa Teresa | Ruschi^39^ reported occurrences at the Santa Lúcia Ecological Station, in Santa Teresa municipality, between 1939 and 1976. The station is located approximately 100 km southwest of the VNR and SBR. | Ruschi 1977 |
| 1940 |  | Santa Teresa | Ruschi^40^ reported a specimen collected in 1940 (MBML 6762), in Santa Teresa municipality. The municipality is located approximately 100 km southwest of the VNR and SBR. A juvenile male Harpy Eagle^61^. | Ruschi 1979 |
| 10/06/1941 |  | Colatina | A specimen housed at the National Museum of Rio de Janeiro, collected in Colatina municipality on June 10, 1941 (MN 39629/MF 108). The municipality is located approximately 60 km southwest of the VNR and SBR. An adult male Harpy Eagle^61^. | MN 39629 |
| 1944 |  | Linhares | Ruschi^40^ reported a nest in the municipality of Linhares in 1944, in a *Cariniana legalis* tree. Since the reserves are located within the municipality, the nest could have been situated either within or up to 50 km away from the VNR and SBR. | Ruschi 1979 |
| 1944 |  | Santa Teresa | Ruschi^40^ reported a specimen collected in 1944 and kept in captivity until 1945 (MBML 2097), in Santa Teresa municipality. The municipality is located approximately 100 km southwest of the VNR and SBR. An adult female Harpy Eagle^61^. | Ruschi 1979 |
| Before 1946 |  | São Gabriel da Palha | Ruschi^40^ reported a nest in São Gabriel da Palha municipality, in a sapucaia tree (*Lecythis urnigera*). An eaglet was reportedly collected after falling from the nest. The municipality is located approximately 15 km north of the VNR and SBR. | Ruschi 1979 |
| Before 1946 | Córrego do Veado Biological Reserve | Pinheiros | Ruschi^40^ reported a sighting in the Córrego do Veado Biological Reserve, Pinheiros municipality, located approximately 60 km north of the VNR and SBR. | Ruschi 1979 |
| Before 1946 | Claro River | Iúna | Ruschi^40^ reported a collected individual in Rio Claro, Iúna municipality, approximately 200 km southwest of the VNR and SBR. | Ruschi 1979 |
| 1953 |  | Colatina | A specimen collected in Colatina in 1953, initially housed at the Itatiaia Regional Museum of Fauna and Flora (MRFF 424) and later transferred to the National Museum of Rio de Janeiro (MN 44680). The municipality is located approximately 60 km southwest of the VNR and SBR. A female Harpy Eagle^61^. | MN 44680 |
| 1957 |  | Domingos Martins | Ernande Ewald presented a Harpy Eagle talon from an individual that was shot in 1957 by his father, Henrique Ewald, on the family property in the district of Panelas, in the municipality of Domingos Martins. The killing was reportedly motivated by fear that the eagle might attack family members. Approximately 150 km southwest of the VNR and SBR. | Ernande Ewald (pers. comm.) |
| 1958 |  | Rio Bananal | A specimen (ML 0361) mounted at the Lorenzutti Museum in Linhares in 1958, with a label indicating Colatina municipality as the origin. However, according to Emiliano Bonfá (pers. comm.), the bird was shot on a rural property in Rio Bananal municipality. The municipality is located approximately 25 km from the VNR and SBR. A female Harpy Eagle^61^. | ML 0361 |
| Late 1960s | Blay Stream | São Gabriel da Palha | Luiz Leôncio Lorenzoni presented a photo and a piece of skin from a Harpy Eagle’s claw from an individual that was shot on a rural property in Córrego Blay, in São Gabriel da Palha municipality, in the late 1960s. Approximate coordinates: 19°01'54.7"S 40°28'40.3"W. Approximately 25 km north of the VNR and SBR. The claw is currently in the possession of Amarildo Lorenzoni, a local of the region. A female Harpy Eagle^61^. | Luiz Leôncio Lorenzoni (pers. comm.) |
| Late 1960s | Blay Stream | São Gabriel da Palha | Luiz Leôncio Lorenzoni reported a second individual that was shot on a rural property in Córrego Blay, in the municipality of São Gabriel da Palha, in the late 1960s. Approximate coordinates: 19°01'54.0"S 40°29'00.9"W. Approximately 25 km north of the VNR and SBR. The carcass was reportedly sent to a local Combonian Catholic seminary for taxidermy, but no further information about its whereabouts was found. | Luiz Leôncio Lorenzoni (pers. comm.) |
| 1968–1969 | Professor Mello Leitão Biology Museum | Santa Teresa | Ruschi^38^ reported the species at the Professor Mello Leitão Biology Museum headquarters, in Santa Teresa municipality, between 1968 and 1969. The museum is located approximately 100 km southwest of the VNR and SBR. | Ruschi 1969 |
| Late 1970s – 24/07/2021 | Surroundings of the Sooretama Biological Reserve | Jaguaré | Two records not compiled in Appendix 1 of this study. Approximately between 0 and 2 km from VNR and SBR. | Fabres et al. (this study) |
| 1981 – 18/01/ 2026 | Sooretama Biological Reserve and surrounding areas | Sooretama | Eighteen records not compiled in Appendix 1 of this study. Approximately between 0 and 2 km from VNR and SBR. | Fabres et al. (this study) |
| 12/03/1970 |  | Linhares | Ruschi^40^ reported a specimen collected in Linhares on March 12, 1970 (MBML 6761), housed at the Professor Mello Leitão Biology Museum (MBML), Santa Teresa municipality. An adult female Harpy Eagle^61^. Approximately between 0 and 50 km from VNR and SBR. | Ruschi 1979 |
| 01/1985 – 09/03/2025 | Sooretama Biological Reserve, Vale Natural Reserve and surrounding areas | Linhares | Sixty-eight records not compiled in Appendix 1 of this study. Approximately between 0 and 2 km from VNR and SBR. | Fabres et al. (this study) |
| 1986 | Córrego Grande Biological Reserve | Pedro Canário | Pacheco et al.^48^reported a sighting in the municipality of Pedro Canário in 1986. Carlos Eduardo de Souza Carvalho (pers. comm.), who made the record, stated that it was at Fazenda Klabin, which is now part of the Córrego Grande Biological Reserve. The Reserve is located approximately 60 km north of the VNR and SBR. | Pacheco et al. 2003 |
| 29/06/1990 | Augusto Ruschi Biological Reserve | Santa Teresa | Pacheco et al.^48^reported the first sighting at Nova Lombardia (now Augusto Ruschi Biological Reserve), Santa Teresa municipality. The Reserve is located approximately 100 km southwest of the VNR and SBR. | Pacheco et al. 2003 |
| 1990 | Augusto Ruschi Biological Reserve | Santa Teresa | Pacheco et al.^48^ reported a second sighting at Nova Lombardia (Augusto Ruschi Biological Reserve), Santa Teresa municipality, in 1990. The Reserve is located approximately 100 km southwest of the VNR and SBR. | Pacheco et al. 2003 |
| 2000-2004 |  | Linhares | Roberto Azeredo (pers. comm.) reported that an individual rescued in the municipality of Linhares was sent to Crax – Wildlife Research and Conservation Society, in Contagem, Minas Gerais. Approximately within 50 km from VNR and SBR. | Roberto Azeredo (pers. comm.) |
| 07/04/2009 | Augusto Ruschi Biological Reserve | Santa Teresa | Novaes et al.^52^ recorded the first Harpy Eagle vocalization at the Augusto Ruschi Biological Reserve. The Reserve is located approximately 100 km southwest of the VNR and SBR. | Novaes et al. 2010 |
| 13/07/2009 | Augusto Ruschi Biological Reserve | Santa Teresa | Novaes et al.^52^ made a second vocalization recording at the Augusto Ruschi Biological Reserve. The Reserve is located approximately 100 km southwest of the VNR and SBR. | Novaes et al. 2010 |
| 01/09/2012 | Augusto Ruschi Biological Reserve | Santa Teresa | Romenique Raton^55^ recorded a photo observation on September 1, 2012, at the Augusto Ruschi Biological Reserve. The Reserve is located approximately 100 km southwest of the VNR and SBR. | Raton 2012 |
| 2013 | Maria Bonita Farm | Linhares | Letícia Magnago (pers. comm.) recorded a vocalization in April 2013 at Maria Bonita Farm, left north bank of the Doce River, near Lagoa das Palmas, Linhares municipality. Approximate coordinates: 19°27'17.5"S 40°12'30.3"W. Approximately 30 km southwest of the VNR and SBR. | Letícia Magnago (pers. comm.) |
| 03/2018–27/03/2026 | Mountain Zoo Park | Marechal Floriano | Between March 2018 and March 2026, birdwatchers and staff from Mountain Zoo Park (Elmir Rodrigues, pers. comm.) recorded over 46 visits by a wild adult female Harpy Eagle to the zoo in Marechal Floriano. The zoo is located approximately 150 km southwest of the VNR and SBR. | Magnago 2018; Elmir Rodrigues (pers. comm.) |
| 01/02/2026 | Parque das Hortênsias Condominium, in the district of Soído | Domingos Martins | Adalberto Ramaldes and Cássia Araújo Ramaldes photographed an adult Harpy Eagle in the afternoon of February 1, 2026, from their backyard at Parque das Hortênsias Condominium, in the district of Soído. Approximately 150 km southwest of the VNR and SBR. | Ramaldes 2026 |
